# Detecting virus integration sites based on multiple related sequencing data by VirTect

**DOI:** 10.1186/s12920-018-0461-8

**Published:** 2019-01-31

**Authors:** Yuchao Xia, Yun Liu, Minghua Deng, Ruibin Xi

**Affiliations:** 1 School of Mathematical Sciences, Peking University, Beijing, 100871 China; 2 Center for Quantitative Biology, Peking University, Beijing, 100871 China; 3 Center for Statistical Science, Peking University, Beijing, 100871 China; 4 Center for Data Science, Peking University, Beijing, 100871 China

**Keywords:** Hidden Markov model, Split reads, Paired-end reads, HBV, HPV

## Abstract

**Background:**

Since tumor often has a high level of intra-tumor heterogeneity, multiple tumor samples from the same patient at different locations or different time points are often sequenced to study tumor intra-heterogeneity or tumor evolution. In virus-related tumors such as human papillomavirus- and Hepatitis B Virus-related tumors, virus genome integrations can be critical driving events. It is thus important to investigate the integration sites of the virus genomes. Currently, a few algorithms for detecting virus integration sites based on high-throughput sequencing have been developed, but their insufficient performance in their sensitivity, specificity and computational complexity hinders their applications in multiple related tumor sequencing.

**Results:**

We develop VirTect for detecting virus integration sites simultaneously from multiple related-sample data. This algorithm is mainly based on the joint analysis of short reads spanning breakpoints of integration sites from multiple samples. To achieve high specificity and breakpoint accuracy, a local precise sandwich alignment algorithm is used. Simulation and real data analyses show that, compared with other algorithms, VirTect is significantly more sensitive and has a similar or lower false discovery rate.

**Conclusions:**

VirTect can provide more accurate breakpoint position and is computationally much more efficient in terms both memory requirement and computational time.

**Electronic supplementary material:**

The online version of this article (10.1186/s12920-018-0461-8) contains supplementary material, which is available to authorized users.

## Background

Cancer is a very heterogeneous disease. Tumor genomes can be vastly different between and, probably more importantly, within cancer patients. The intra-tumor heterogeneity poses great challenges for tumor treatments. In recent years, multi-regional high-throughput sequencing (HTS) has been widely used for studying intra-tumor heterogeneity [1–4], where tumor cells from different tumor regions are sequenced and somatic variations are profiled. These studies revealed that the level of intra-tumor heterogeneity varies greatly between different types of tumors and between different tumor patients. Because of intra-tumor heterogeneity, drug responses of tumor cells from different regions can also be significantly different [5]. Recently, joint somatic mutation detection algorithms based on multi-regional sequencing were developed [6]. These algorithms can greatly increase the sensitivity of somatic mutation detection and hence can provide more accurate mutation data for tumor heterogeneity and tumor evolution studies.

Many human cancers (~ 10–15%) are caused by viruses [7] such as human papillomavirus (HPV) [8] and Hepatitis B Virus (HBV) [9]. Viruses such as HPV and HBV can integrate their genomes to their host genomes and this genome integration is believed to be the major mechanism for their carcinogenic effects [10, 11]. Accurate detection of virus integration sites can provide invaluable information for studying molecular mechanisms of virus-related cancers, cancer genome evolution and even for developing cancer treatments. Recently, a number of algorithms have been developed for detecting viruses based on cancer sequencing data [12–16]. Several of them are designed to detect virus integration sites. For example, Virana [13] and VirusSeq [12] can detect virus integration sites based on whole transcriptome sequencing (RNA-Seq) data. ViralFusionSeq [17] and VirusFinder [18, 19] can be used for whole-genome sequencing (WGS), whole exome sequencing (WES) data as well as for RNA-Seq data. However, the sensitivity of these methods is still low. When applying these methods to multi-regional sequencing data, because of their low sensitivity, common integration sites can very likely be detected in only a few regions but not in all regions. These false negatives can lead to over-estimation of tumor heterogeneity and incorrect inference of tumor evolution. Although increasing sequencing coverage could ameliorate this problem, it will also significantly increase the experimental costs. In addition, current methods are computationally very expensive in terms of memory requirement and computational time, making it very difficult to apply them to high coverage whole genome sequencing data.

In this paper, we introduce VirTect for sensitive and accurate detection of virus integration sites from multi-related-sample HTS data. VirTect makes full use of HTS data from multiple samples without pooling the data together and performs integrated analysis to detect virus integration sites. Compared with available virus detection methods, VirTect is significantly more sensitive and can provide more accurate breakpoint position with similar or lower false discovery rate (FDR). VirTect is computationally much more efficient than other algorithms in terms of both computational time and memory requirement—it only needs around one fifth of computational time of other methods. Furthermore, since VirTect performs joint analysis of multiple sample data, VirTect will give exactly the same breakpoint estimate for shared integration sites among different samples, and thus subsequent analysis such as tumor heterogeneity analysis and tumor evolution analysis would be more convenient.

## Methods

**Fig. 1:**
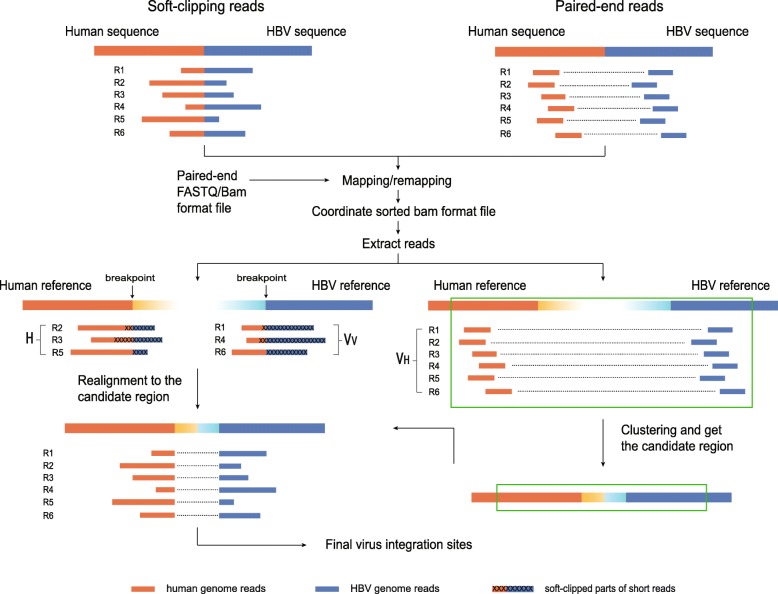
The overall workflow of VirTect. After mapping or remapping of short reads to the human and virus genome, VirTect extracts short reads that might contain virus integration information for further analysis. The reads whose one ends were mapped to the human genome and other ends were mapped to the virus genome are clustered to get the candidate integration regions (bottom right). The reads that are soft-clippedly mapped to the human or the virus genome are realigned to candidate regions to get the exact breakpoints (bottom left). The soft-clipped parts of the short reads are marked with X’s

The overall workflow of VirTect is shown in Fig. 1. VirTect uses fastq files or bam files of paired-end reads data as input. Fastq or bam files from different samples do not need to be merged as a single file and VirTect automatically extract necessary information. VirTect first aligns (for data in fastq files) or realigns (for data in bam files) short reads to human and virus reference genomes. Since the samples are related tumor samples, a portion of the virus integrations should be shared among some or all samples. Therefore, if all samples are pooled together, on average, the shared integrations would have more supporting reads than private integrations. Hence, the detection power for shared integration should be higher than private integrations. However, physically pooling all data together would be computationally not efficient. Instead, after mapping for each individual sample, VirTect extracts all reads from all samples that might contain virus integration information and use these reads jointly to detect integrations. These potential supporting reads are paired-end reads partially mapped to virus genomes or soft-clippedly mapped. Then, VirTect performs joint clustering analysis and joint precise local realignment of the extracted reads. Each cluster corresponds to one candidate virus integration site. A local precise hidden Markov Model (HMM) realignment procedure is applied to the reads in each cluster to get accurate integration sites. Details of VirTect are described below.

### Data preprocessing

VirTect can use either raw data in fastq format as input or BWA-aligned [20] data in bam format as input. If users choose to use raw data as input, VirTect first maps the paired-end read to the host reference genome (e.g. human reference genome) and virus genomes using BWA. Samtools [21] are used to sort the bam files and remove duplicate reads. If the data are in bam format, we assume that short reads are only mapped to the host reference genome. In this case, VirTect first extracts all partially unaligned short reads and realign those reads to the host reference genome and the virus genome using BWA. We consider the bam format input because researchers often have bam files available for detecting other types of genomic variations. Starting from bam files, VirTect can save a significant amount of computational time. The virus genome usually can be obtained from databases such as (1) the NCBI virus database (ftp://ftp.ncbi.nlm.nih.gov/blast/db/), (2) Genome Information Broker for Virus database (http://www.insdc.org/) [12], or (3) the virus database used by RINS [15].

VirTect extracts three sets of paired-end reads *V*_*V*_, *V*_*H*_ and *H* from all related samples (Fig. 1). A paired-end read belong to *V*_*V*_ if its both ends are mapped to the virus genomes but at least one end is soft-clipped. If one end of a read pair is mapped to the host genome and the other end to the virus genomes, we denote this read pair belonging to *V*_*H*_. The read set *H* consists of paired-end reads whose both ends are mapped to the host genome, but whose one ends are soft-clipped. Note that the short read sets *V*_*V*_, *V*_*H*_ and *H* do not overlap and all the following analyses are based on these three sets of reads.

### Virus integration sites detection

Given all reads in *V*_*V*_, *V*_*H*_ and *H* , VirTect first clusters reads in *V*_*H*_ to get potential integration sites, and then applies a precise local realignment procedure to reads in *V*_*V*_ and *H* to get the exact breakpoint of the integration sites. In the clustering step, all reads in *V*_*H*_ are first sorted by their mapping coordinates on the host genome. VirTect employs an iterative procedure to cluster these reads and each cluster corresponds to a potential integration site. VirTect performs clustering for each chromosome of the host genome separately. Below we use one read to refer to one paired-end read (including both of its ends). Given a chromosome A in the host genome, suppose that ${V}_H^A\subset$ *V*_*H*_ is the set of reads whose one end is mapped to chromosome A and the other end is mapped to the virus genome. For any two pairs of short reads in ${V}_H^A$, we define their distance as the absolute difference of their mapping coordinates on chromosome A. The distance between two sets of short reads *C*_1_ and *C*_2_ is defined as the minimum distance between pairs of short reads in C_1_ and *C*_2_. We cluster short reads in ${V}_H^A$ to M clusters *C*_1_, ⋯, *C*_*M*_ such that the distance between any two clusters is larger than a threshold *T* and each cluster cannot be further divided into two clusters with their distance greater than *T*. This is achieved by first sorting short reads in ${V}_H^A$ increasingly by their mapping positions on chromosome A. Then, VirTect puts the first short read (i.e. the short read with the smallest mapping coordinate in A) as the first cluster *C*_1_. Suppose that after *k* steps, we obtain *m* clusters *C*_1_, ⋯, *C*_*m*_. At the k + 1th step, VirTect takes the k + 1th read pair *R*_*k* + 1_ and calculates its distances *d*_*m*_ to the cluster *C*_*m*_. If *d*_*m*_ is less than the threshold *T*, we assign this read *R*_*k* + 1_ to the cluster *C*_*m*_; Otherwise, we assign the read *R*_*k* + 1_ to a new cluster *C*_*m* + 1_. Note that since all paired-end reads are sorted increasingly by their mapping coordinates, the clusters *C*_*i*_ (*i* = 1, ⋯, *m*) are also sorted increasingly. The distance between *R*_*k* + 1_ and the cluster *C*_*m*_ will be the smallest among the distances between *R*_*k* + 1_ and the clusters *C*_*i*_ (*i* = 1, ⋯, *m*). Therefore, *d*_*m*_>T implies that all distances between *R*_*k* + 1_ and *C*_*i*_ (*i* = 1, ⋯, *m*) is greater than *T*. By default, the threshold *T* is chosen as the mean insert size plus three standard deviations of the insert sizes.

Given a cluster *C* obtained from the above step, suppose that *M*_1_ and *M*_2_ are the minimum and maximum of the mapped coordinate positions in the host genome of the reads in the cluster *C*. Suppose that *S*_*h*_ (the subscript *h* refers to the host genome) is the subsequence of the host reference genome from *M*_1_ − *N* to *M*_2_ + *N* (*N* is taken as 500 by default). Then, *S*_*h*_ is our candidate virus integration region. Assume for now that each read in the cluster *C* have one end mapped to the host genome and the other end mapped to the same virus genome. Using the same procedure, we could also get a subsequence *S*_*v*_ (the subscript *v* refers to the virus genome) from the virus reference genome. VirTect concatenates the sequence *S*_*h*_ and *S*_*v*_ and get a new sequence *S*. This new sequence *S* is constructed for our precise local realignment. Then, VirTect selects reads in *H* that are soft-clippedly mapped to *S*_*h*_ and all reads from *V*_*V*_ for precise local HMM realignment [22]. Denote this set of reads as *U*. Since there is a large gap in *S*, a sandwich realignment [23] is used to realign the soft-clipping reads to the reference sequences based on the HMM realignment algorithm described in Xia et al. 2017 [24]. In the sandwich realignment, short reads are realigned both from the 5` end and the 3` end, which thus allows VirTect utilizing both the non-clipped and the clipped part of the short reads for precise localization of the breakpoints. The HMM model used in the sandwich realignment has an end-of-mapping state. With this state, the HMM realignment will not try to map every base pairs of short reads. Instead, it will terminate the mapping at or near the breakpoint, because after the breakpoints the mapping will contain many mismatches and/or gaps. Another advantage of this sandwich realignment is that if there are micro-insertions or micro-insertions at breakpoints, the mapping will automatically terminate and the split positions of short reads are naturally determined. The sandwich realignment does not need to explicitly search for the best split position. After realignment, VirTect first filters short reads whose either of its two directional mappings has less than 10 consecutive matches. VirTect also filters reads whose both directional mappings are mapped to the same side of junction point of *S* (i.e. the junction between *S*_*h*_ and *S*_*v*_), because these reads do not span the breakpoint and cannot be used for breakpoint estimation. For the mappings to the left of the junction point of *S* (i.e. the mappings are to *S*_*h*_), we take the median of the ending positions of the mappings as the estimate of the integration site on the host genome. The breakpoint on the virus genome is estimated by the median of the ending positions of the mappings to the right of the junction point of *S* (Fig. 1.). Lastly, VirTect will call this candidate region as an integration site if there are at least two reads whose sandwich alignments support the integration. All samples having paired-end alignments or sandwich alignments supporting the integration event are predicted to harbor this integration event.

If the reads in the cluster are mapped to a few different virus genomes (one end of the read always map to the host genome, but the other end map to different virus genomes), we create a concatenated sequence *S* for each mapped virus genome. For all soft-clippedly mapped to *S*_*h*_, we perform sandwich realignment to each concatenated sequence *S*. We then can obtain a realignment likelihood for each concatenated sequence *S* and choose the virus genome with the largest likelihood for further analysis. If two likelihoods are the same, we choose the virus genome having the most number of reads in the cluster for further analysis.

## Results

### Simulation study

We first compare the performance of VirTect with other three methods ViralFusionSeq, VirusFinder2 and Virus-Clip [25] using simulation. We randomly select 160 viral sequences (sizes ranging from 500 bp to 1000 bp) from genotype C [26] of the HBV genome and insert them to chromosome 1, 2, 3 and 4 of the human reference genome (hg19, GRCh37). The GenBank ID of HBV Genotype C is AB014381.1. In this way, we generate five related genomes with virus integrations. Each genome has 40 virus integration sites and 25 of them are common in all five genomes. In the simulation, we also randomly put SNVs and Indels near the integration site (50 bp neighborhood). Given the four simulated genomes, we use ART [27] to simulate the Illumina paired-end reads with a read length of 100 bp and an insert size of 300 bp (standard deviation 50 bp). For each genome, we simulate six datasets at coverage 3X, 5X, 10X, 20X, 30X and 40X. For VirTect, we test its performance starting from fastq files (VirTect:fastq) and from bam files (VirTect:bam). For VirTect:fastq, we use BWA to map all paired-end reads to the human reference genome (hg19) and the HBV genomes (genotype A-H) simultaneously. For VirTect:bam, the short reads are first mapped to the human reference genome. VirTect then uses BWA to realign the partially unaligned reads to the HBV genomes (genotype A-H). For the other two algorithms, we use the default parameter settings. All algorithms are tested on a Linux sever (32-core Intel Xeon 2.40 GHz CPU and 256Gb memory).

**Fig. 2:**
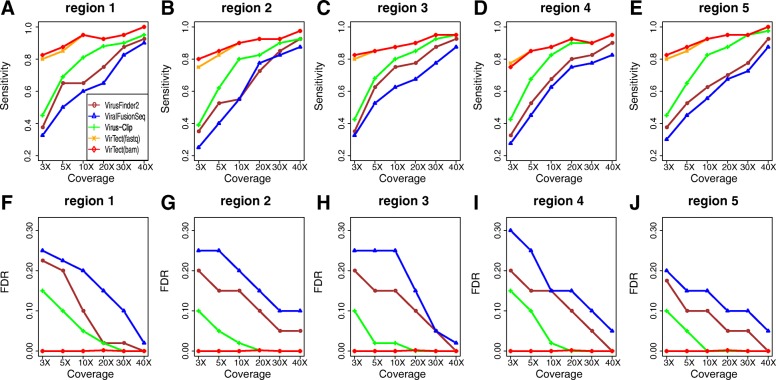
The sensitivity (**a**-**e**) and FDR (**f**-**j**) of the four algorithms on the simulation data at different sequencing coverages

We first apply the other three algorithms to each data set individually and compare their performances with VirTect. Figure 2 shows the sensitivities and false discovery rates (FDR) of these algorithms on each genome separately. We define an integration prediction as a true positive if the distance between the predicted integration site and the real integration site is less than 350 bp. We find that VirTect achieves the highest sensitivities and the lowest FDR across all five genomes. Especially, at low coverage depth (3X, 5X and 10X), the sensitivities of VirTect are much higher than the other three algorithms and its FDRs are 0. VirTect:fastq is a little more sensitive than VirTect:bam at 3X and 5X coverage, but overall their performances are very similar. The other three algorithms had a higher FDR at low coverage because a number of predicted integration sites are far from the true integration sites.

**Fig. 3:**
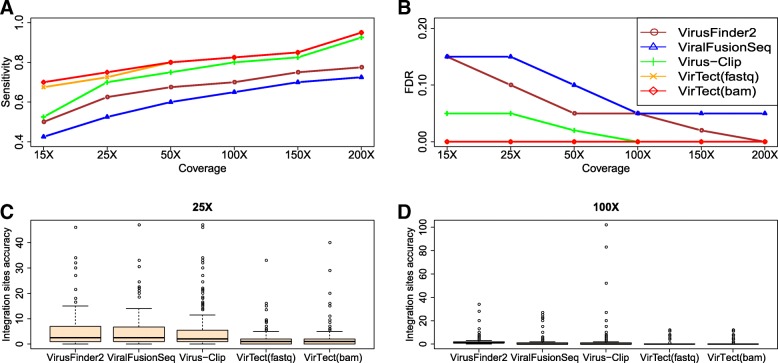
The Sensitivity (**a**) and FDR (**b**) on the merged data. (**c**, **d**) Boxplots of breakpoint estimation accuracy on merged data at 25X and 100X coverage

**Fig. 4:**
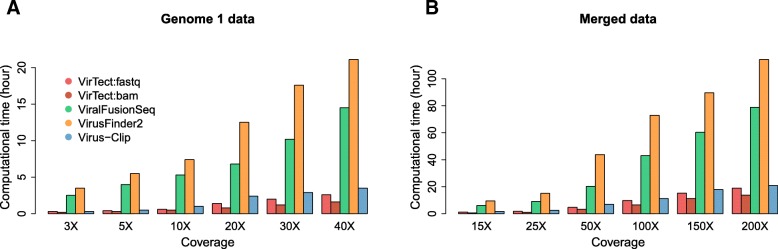
**a** The computational time (in hour) on the simulated Genome 1 data at different coverages. **b** The computational time (in hour) on merged data at different coverages

The above comparison is a bit unfair for other algorithms because the other three algorithms do not use all data to detect common integration sites. We then merge sequencing data of the five genomes as one data set and apply the other two algorithms to the merged data and compare their performances. Note that for VirTect, we do not need to physically merge the data and this is more convenient to analyze multiple related-samples. Figure 3a-b shows that VirTect also has the highest sensitivity and the lowest FDR across all coverages among the three algorithms. Figure 3c and d shows the distance between the detected integration sites and the true integration sites at 25X and 100X coverage. Compared with the other algorithms, the integration sites predicted by VirTect are closest to the true integration sites and the predicted integration sites of VirTect are only up to a few bp away from the true integration sites in most cases. We also compare the computational time of different algorithms. Figure 4. shows the running time using eight cores on the simulation dataset of Genome 1 and the merged dataset, respectively. We see that VirTect only takes around 1 fifth of the computational time of ViralFusionSeq and VirusFinder2 and a little faster than Virus-Clip.

### Real data analysis

In this section, we compare the performance of VirTect with the other two algorithms on real data sets. We consider two real data sets in this study. One is a multi-regional whole exome sequencing (WES) data from an HBV-related hepatocellular carcinoma (HCC) patient [28]. The patient’s ID is 213 and tumors from five regions are sequenced by Illumina platform with a read length of 75 bp. The mean insert size is 200 bp with a standard deviation of 50 bp. The other data consists of nine whole genome sequencing (WGS) data of HBV-related HCC patients [29]. The read length of this data is 90 bp and the coverage is around 30X.

**Fig. 5:**
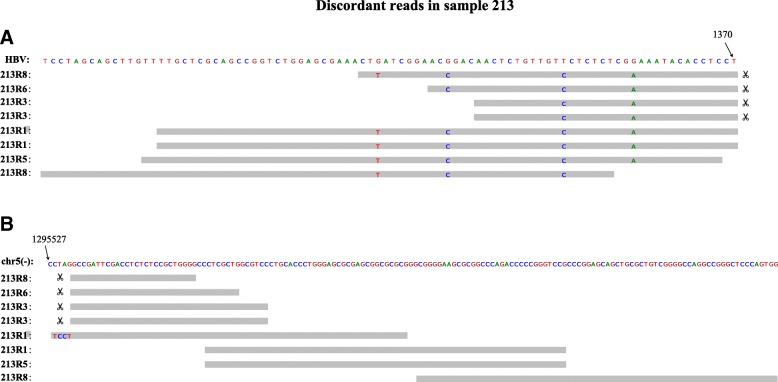
VirTect identifies an HBV integration site at the *TERT* promoter region in patient 213. All tumors from different regions have this integration event. The discordant and sandwich-mapped reads to the HBV genome (**a**) and human genome (**b**) are shown

For the multi-region WES data, VirTect is able to detect one HBV integration sites. The integration site is at chromosome 5:1295527 (Fig. 5). The integration sites are located at promoter region of the telomerase reverse transcriptase (TERT). Previous research showed that TERT is the most prevalent gene integrated by HBV in HCC [30]. Moreover, all tumor regions have this integration event, implying that this event might be an early carcinogenesis event. When we apply the other two algorithms to data of each region, they fail to detect any integration site. When we merge the multi-regional data together, they also fail to detect any event.

**Fig. 6:**
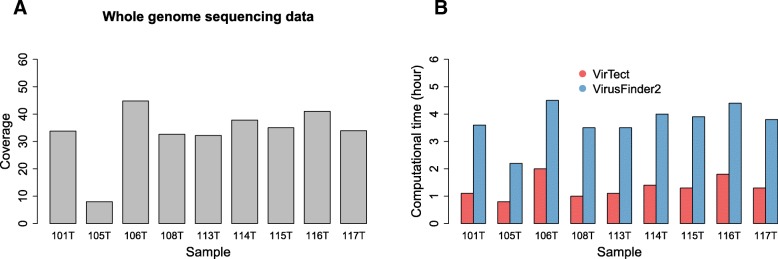
**a** The mean coverage of the 9 WGS data. **b** The computational time (in day) of VirTect and VirusFinder2 on these 9 WGS data

**Fig. 7:**
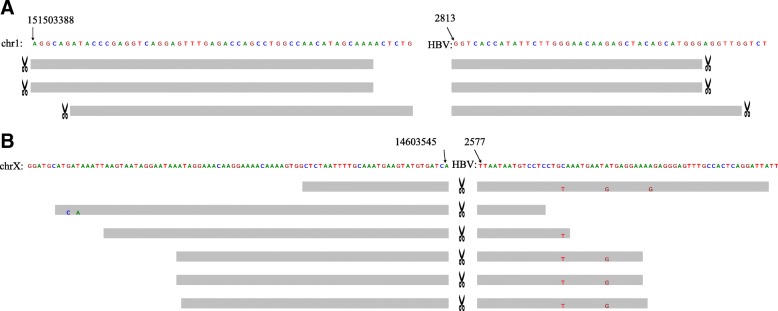
**a** A known HBV integration site detected by both VirTect and VirusFinder2 in sample 101 T. The mappings of the supporting reads to the human genome (left panel) and to the virus genome (right panel) are shown. The split position of each read is marked by a scissor icon. **b** A new integration site detected by VirTect

For the WGS data, we downloaded hepatocellular carcinoma samples, 101 T, 105 T, 106 T, 108 T, 113 T,114 T,115 T,116 T and 117 T reported by Sung et al. 2012 [29]. Here, we only report the results for VirTect and VirusFinder2 because ViralFusionSeq failed due to insufficient memory and Virus-Clip did not finish computation after a week. The running time of VirTect and VirusFinder2 is shown in Fig. 6. VirTect and VirusFinder2 detected all integration sites reported by Sung et al. 2012 [29]. Some of these integration sites interrupt important cancer genes such as CCNE1 (sample 106 T, chr19:30304177) and NTRK3 (sample 108 T, chr15:88688212). Details about these integration sites are in Additional file 1: Table S1. Figure 7a shows one integration cite at chr1:151503388 at the gene CGN. VirusFinder2 costs long time (> 3 days) and a large amount of memory (about 70 Gb) to finish the computation. In comparison, VirTect uses 1.5 days and no more than 30Gb memory. In addition to the reported integration sites, VirTect detects a new integration site at chromosome X:14603545 (Fig. 7b) overlapping with the gene GLRA3.

## Discussion

To our knowledge, VirTect is the first algorithm capable of detecting virus integration from multiple related tumor samples. Intra-tumor heterogeneity and tumor cell evolution have received great research attention recently. Virus integration site can provide valuable information for inferring the evolution history of tumor cells. For example, if all tumor cells have exact the same integration site, we can confidently infer that these tumor cells evolve from the same ancestor. VirTect could also be used to detect virus integration in RNA-seq data, especially multiple-related RNA-seq data. One drawback of VirTect is that it requires known viruses. Hence, VirTect is not suitable to detect integration sites of unknown viruses.

## Conclusion

In this paper, we develop a computational tool called VirTect for virus integration site detection. Simulation and real data analysis show that VirTect performs considerable better than other available algorithms in terms of sensitivity, false discovery rate, breakpoint position, computational time and memory requirement. With its high integration detection accuracy, we expect that VirTect can be widely applied to virus integration genomics studies.

## Availability and requirements

**Project name:** VirTect.

**Project home page:** https://github.com/xyc0813/VirTect/

**Operating system(s):** Windows,Unix-like (Linux, Mac OSX).

**Programming language:** python(> = 2.7),Cython.

**Any restrictions to use by non-academics:** None.

## Additional File

Additional file 1: Table S1. The integration cites detected by VirTect and VirusFinder2 in the nine whole genome sequencing data of HBV-related HCC patients. (XLS 30 kb)

## Abbreviations

HBV: Hepatitis B Virus
HPV: Human papillomavirus
HTS: High-throughput sequencing
WES: Whole exome sequencing
WGS: Whole genome sequencing
